# Supplementary figures and images for: Human Health Benefits of Non-Conventional Companion Animals: A Narrative Review
Source: Animals (Basel). 2022 Dec 21;13(1):28. doi: 10.3390/ani13010028 (PMC9817996; doi:10.3390/ani13010028)

## Identification of studies via databases and registers

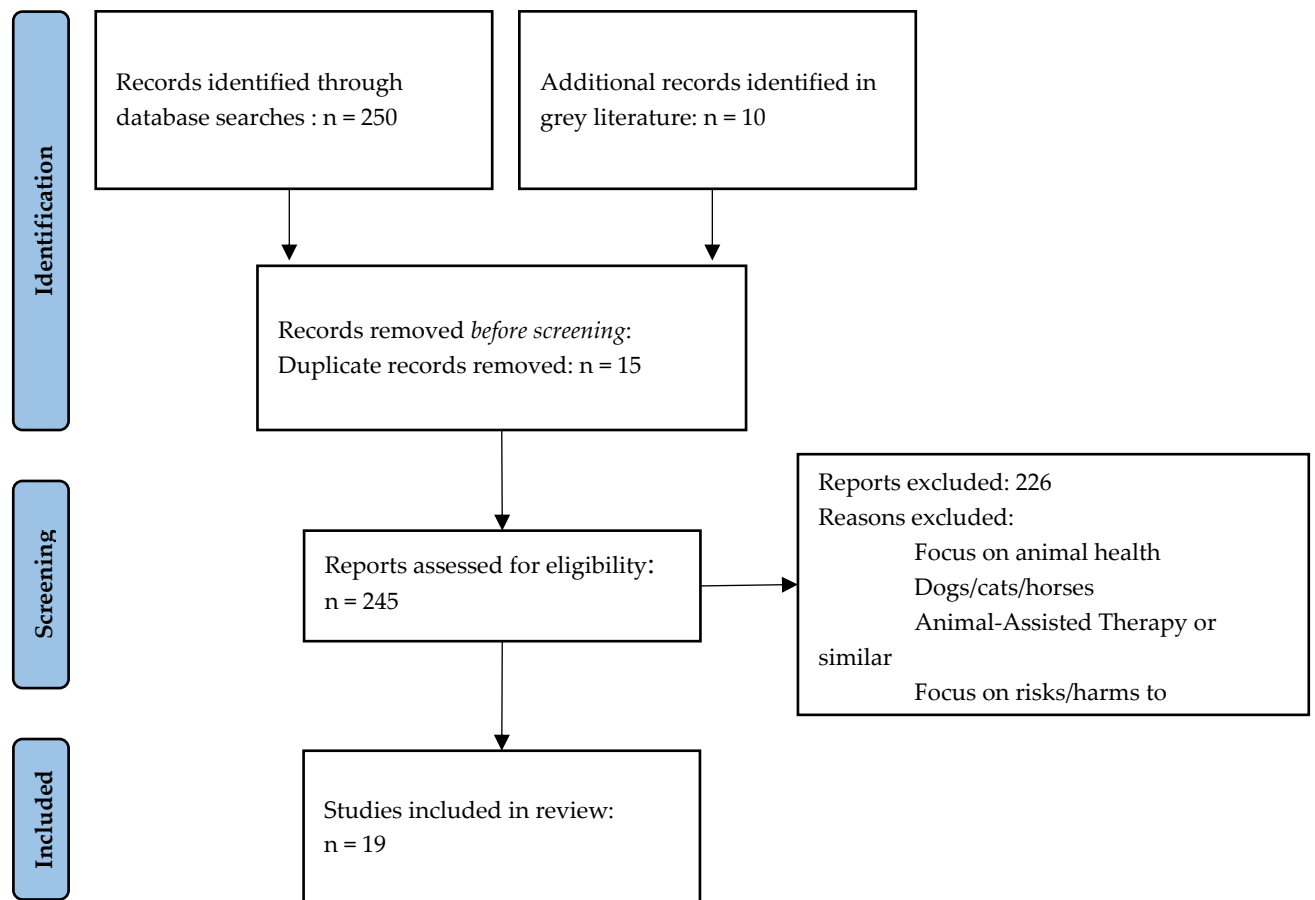

Supplement: Supplementary file 1 [file animals-13-00028-s001.zip › animals-2070887-supplementary.pdf]
